# Differential lung NK cell responses in avian influenza virus infected chickens correlate with pathogenicity

**DOI:** 10.1038/srep02478

**Published:** 2013-08-21

**Authors:** Christine A. Jansen, Eveline D. de Geus, Daphne A. van Haarlem, Peter M. van de Haar, Brandon Z. Löndt, Simon P. Graham, Thomas W. Göbel, Willem van Eden, Sharon M. Brookes, Lonneke Vervelde

**Affiliations:** 1 Department of Infectious Diseases and Immunology, Faculty of Veterinary Medicine, Utrecht University, Utrecht, the Netherlands; 2 Department of Virology, Animal Health and Veterinary Laboratories Agency (AHVLA) Weybridge, Addlestone, Surrey, United Kingdom; 3 Institute for Animal Physiology, Department of Veterinary Sciences, Ludwig Maximilians University Munich, Munich, Germany; 4 Current address: The Roslin Institute and Royal (Dick) School of Veterinary Studies, University of Edinburgh, Easter Bush, United Kingdom.

## Abstract

Infection of chickens with low pathogenicity avian influenza (LPAI) virus results in mild clinical signs while infection with highly pathogenic avian influenza (HPAI) viruses causes death of the birds within 36–48 hours. Since natural killer (NK) cells have been shown to play an important role in influenza-specific immunity, we hypothesise that NK cells are involved in this difference in pathogenicity. To investigate this, the role of chicken NK-cells in LPAI virus infection was studied. Next activation of lung NK cells upon HPAI virus infection was analysed. Infection with a H9N2 LPAI virus resulted in the presence of viral RNA in the lungs which coincided with enhanced activation of lung NK cells. The presence of H5N1 viruses, measured by detection of viral RNA, did not induce activation of lung NK cells. This suggests that decreased NK-cell activation may be one of the mechanisms associated with the enhanced pathogenicity of H5N1 viruses.

In the immune response against viruses like influenza, NK cells play an important role^1^. NK cells express both activating and inhibitory receptors, and the balance between these signals determines NK-cell activation^2^,^3^. The activating NK-cell receptor NKp46 is mainly expressed on NK cells but has also been reported on a minor fraction of NKT cells^4^ and gamma delta T cells^5^. NKp46 has been demonstrated in different species including humans^6^, monkeys^7^, rodents^8^, cattle^9^, sheep^10^ and pigs^11^. NKp46 and NKp44, another member of the family of natural cytotoxicity receptors, bind viral haemagglutinin (HA) of various strains of influenza and binding results in activation of NK cells^12^,^13^,^14^. *In vivo* studies in mice have shown that NK cells^15^,^16^,^17^ and NKp46^18^ are required for the clearance of influenza virus. In patients with severe influenza infection, diminished frequencies of NK cells are observed in the blood^19^,^20^, and pulmonary NK cells are lacking^21^. This suggests an important role for NK cells in influenza-specific immunity.

Wild aquatic birds are the natural reservoirs for influenza A viruses^22^ which are able to infect both humans and animals and cause seasonal epidemics of infectious respiratory disease in humans worldwide^22^,^23^. These influenza viruses can be characterized based on the antigenic properties of the viral surface proteins HA and neuraminidase (NA)^24^. In birds 16 HA subtypes and 9 NA subtypes have been described^25^. Avian influenza viruses are considered to be of either low pathogenicity or highly pathogenic, based on the ability to induce clinical disease and/or death in chickens^26^. Infection with LPAI virus usually results in mild clinical signs while infection with HPAI viruses induces systemic infection and eventually death of the host within 36–48 hours^27^,^28^. Due to viral mutations these LPAI viruses may give rise to HPAI viruses^29^. Some HPAI viruses cause lethal infection in humans^30^. Also LPAI viruses of the H7 and H9 subtype have been reported to infect humans^31^,^32^,^33^. This makes avian influenza viruses a potential pandemic threat.

The binding of the HA protein to NK cells, similar to the binding of the HA protein to receptors on the host cell, is dependent on sialic acid residues on the NK-cell receptor. The binding of both human and swine influenza viruses to α2,6-linked SA residues on human NKp46^13^ induces NKp46-mediated killing. In contrast, H5N1 HPAI viruses which prefer binding via α2,3-SA residues bind to human NKp46. The interaction between H5N1 virus and NKp46 is not able to induce NK-cell mediated killing by itself. Killing of H5N1 infected targets is only observed when both NKp46 and NKG2D are activated^34^. This lack of NK-cell activation upon the interaction between H5N1 avian influenza viruses and NKp46 itself may be a property of these viruses which contributes to their highly pathogenic nature. Alternatively, it may be caused by the fact that the interactions between avian H5N1 virus and the human NKp46 through its α2,3-SA are insufficient to induce killing by NK cells.

In the present study we hypothesise that the lack of NK-cell activation induced by H5N1 viruses is a property of these viruses, and that the diminished NK-cell activation upon infection with highly pathogenic avian influenza virus is associated with enhanced pathogenicity.

To investigate this, we performed infections in chickens, which can be infected with both LPAI viruses and the deadly HPAI viruses. Studying NK-cell responses in chickens is challenging due to the limited knowledge of non-mammalian NK cells. Avian NK cells have been described as a population of cells which express surface CD8αα homodimers, but no T or B-cell specific antigens^35^. Furthermore, chicken NK cells have been reported to express both activating and inhibitory receptors similar to what has been described in humans^36^,^37^. In a recent study, we identified additional markers that are expressed on chicken NK cells and developed assays to measure NK-cell degranulation and killing^38^.

In the present study activation of lung NK cells was compared following infection with either LPAI or HPAI viruses. Since the role of chicken NK cells upon infection with AIV has not been studied before, we initially studied NK-cell biology upon LPAI infection where we performed a detailed kinetic study. With this knowledge we went on to study NK-cell activation upon HPAI infection. The latter was limited by the timeframe of the infection (birds die within 36–48 hrs).

## Results

### Viral load levels upon infection with LPAI virus

**Figure 1:**
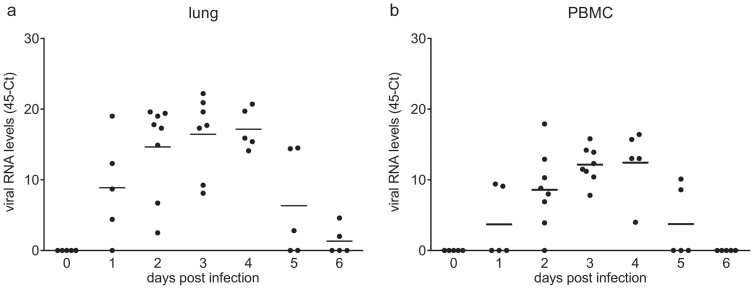
Viral load after infection with LPAI virus. Viral load was determined by qRT-PCR specific for the matrix gene of the influenza virus. Relative expression values were normalized against 28S rRNA. Individual data are shown for lung (a) and PBMC (b) (n = 5; at 2 and 3 dpi n = 8), the bar indicates the mean.

To investigate if infection with LPAI virus would affect multiple lymphoid organs, viral load (RNA level) was determined by qRT-PCR in lungs, blood, and spleen between 0 and 6 days post infection. Virus was detected in the lung at 1 dpi and increased viral loads were seen from 2 dpi onwards (Fig. 1A). This increase continued until 4 dpi, when viral load peaked at a 45-Ct value of 17.2 ± 1.3 (mean ± SEM). At 5 dpi, viral load decreased and was only detectable in two birds at 6 dpi. A similar pattern was observed in PBMC (Fig. 1B). Viral load in PBMC increased up to 4 dpi, but was lower than that in the lung (45-Ct of 12.4 ± 2.2). At 5 dpi viral load declined and was undetectable in all birds at 6 dpi. In spleen, the viral load was very low- the highest viral load was observed at 3 dpi and reached a 45-Ct of 4.8 ± 0.77 (data not shown).

### Characterization of avian NK cells in the lung

**Figure 2:**
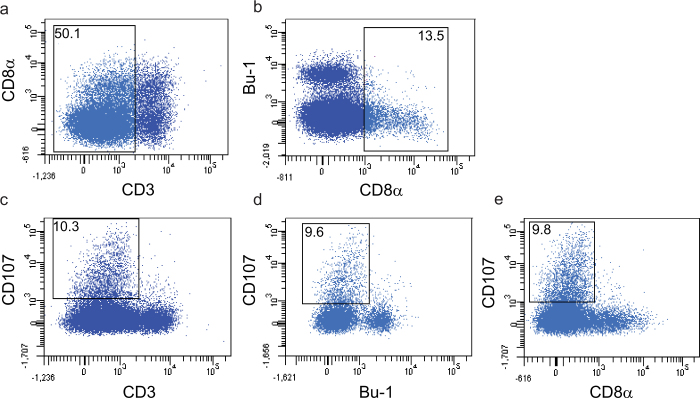
Characterization of avian lung NK cells. Expression of B- and T-cell specific antigens on lung NK cells from an uninfected chicken was analysed by flow cytometry together with the expression of CD107. Since surface CD107 expression was analysed after 4 hours of culture without further stimulation using lung cells of an uninfected bird, the CD107 expression reflects the spontaneous activation of lung NK cells. Cells were gated based on forward-side scatter and dead cells were excluded from the analyses. Co-expression of CD3 and CD8α (a) and the B-cell marker Bu-1 and CD8α (b) was analysed *ex vivo*. Expression of CD107 within CD3 positive and negative populations (c) Bu-1 positive and negative populations (d) and CD3 negative CD8α positive and negative populations (e) was determined after 4 hour incubation at 37°C. Populations of cells showing NK-cell characteristics are indicated.

Avian NK cells have been described as a population of cells which express surface CD8α homodimers, but no T or B-cell specific antigens. These cells have been reported in embryonic splenocytes, in the intestinal epithelium and to a lesser extend in PBMC and spleen^35^,^39^. Since lung NK cells have not been studied before in the chicken, we set out to investigate if lung NK cells can be defined based on the criteria used to describe NK cells in these organs. Therefore expression of B and T-cell specific antigens was analysed together with the expression of CD107 in lung cells of an uninfected bird. Surface expression of CD107 was detected on activated chicken NK cells, similar to what has been described for mammalian NK cells. Since surface CD107 expression was analysed after 4 hours of culture without further stimulation using lung cells of an uninfected bird, the CD107 expression reflects the spontaneous activation of lung NK cells. As shown in Fig. 2, cells expressing surface CD8α and lacking T- or B-cell antigens were observed in lung. CD8α was expressed on both CD3+ and CD3- cells (Fig. 2A), while cells recognized by the B-cell marker Bu-1 lack surface expression of CD8α (Fig. 2B). CD3- cells also lacked surface expression of TCR1, TCR2 and TCR3 (data not shown). Analysis of CD107 expression within these populations of lung cells showed surface CD107 expression on CD3- cells (Fig. 2C), and cells expressing CD107 did not express B-cell antigens (Fig. 2D). Within the CD3- population, cells expressing CD107 did not express CD8α (Fig. 2E). Thus, a population of cells with surface expression of CD8α that lack T and B cell antigens was observed in the lung. Co-staining with mouse-anti-CD107 mAbs showed that these cells that lack B or T cell antigens express CD107, and that CD107 expression was accompanied by loss of CD8α expression.

Since B-cell marker negative cells were either CD3+ or CD3- and as CD107 is known to be expressed by CD3- NK cells and CD3+ cytotoxic T lymphocytes, we defined resting NK cells as CD3-CD8α+ cells (CD8α+ NK cells) and activated NK cells as CD3-CD107+ cells (activated NK cells).

### Frequencies of CD8α+ NK cells in the lung following infection with LPAI virus

**Figure 3:**
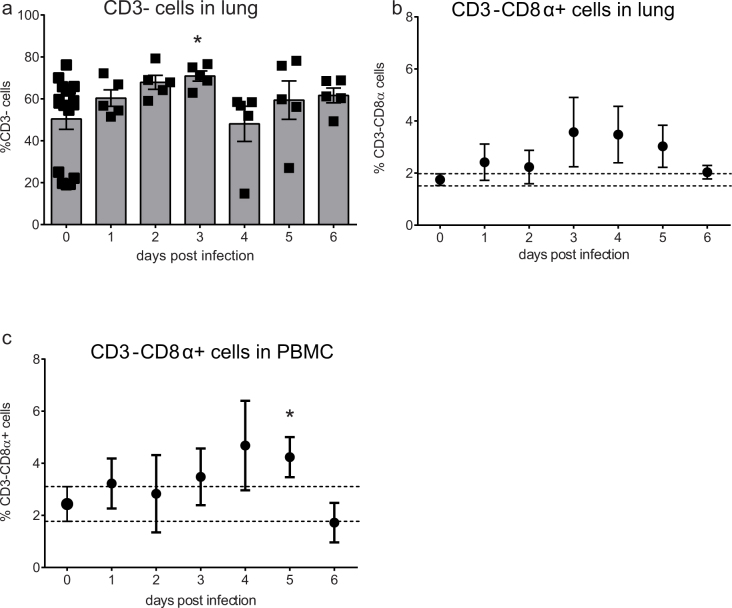
Increased frequencies of lung CD8α+ NK cells 3 days post infection with LPAI virus. Frequencies of lung CD3- cells (a) lung CD8α+ NK cells (b) and blood CD8α+ NK cells (c) were analysed by flow cytometry. Mean ± SEM of five infected birds per day and seventeen uninfected birds (birds from 0 dpi together with the uninfected birds from 1–6 dpi) are shown. Significant differences compared to the uninfected controls were analysed using Mann-Whitney tests and p < 0.05 is indicated by an asterisk.

To study if LPAI virus infection causes a change in the percentage of chicken NK cells in the lung, frequencies of CD3- and CD8α+ NK cells isolated from the lungs were determined between 1–6 dpi. Since we did not observe any difference between the control birds at day 0 and the 2 control birds at the different time points between 1 and 6 dpi, we have combined these birds in one group of uninfected controls. The percentage of CD3- cells increased until 3 dpi when it was significantly higher compared to uninfected controls (70.9 ± 2.4 vs 64.4 ± 1.9 mean ± SEM, Fig. 3A).

Next, the percentage of CD8α+ NK cells were analysed. In uninfected animals, the frequency of CD8α+ NK cells was 1.7 ± 0.2% (Fig. 3B). At 3 dpi the percentage of CD8α+ NK cells in infected animals increased to 3.6 ± 1.3%, approximately 2-fold higher than the proportion of CD8α+ NK cells in uninfected controls. This increase in the percentage of CD8α+ NK cells was maintained at 4 and 5 dpi. At 6 dpi, the percentage of CD8α+ NK cells decreased to 2.0 ± 0.3%, which was similar to the percentage in uninfected controls. The proportion of CD8α+ NK cells in PBMC in uninfected controls (2.4 ± 0.7%) was slightly higher compared to that in lung (1.7 ± 0.2%, Fig. 3D). During infection, the percentage CD8α+ NK cells varied, and only at 5 dpi the percentage of CD8α+ NK cells was significantly higher compared to the uninfected controls (4.2 ± 0.8%, p < 0.05).

### Different populations of lung NK cells after LPAI infection

**Figure 4:**
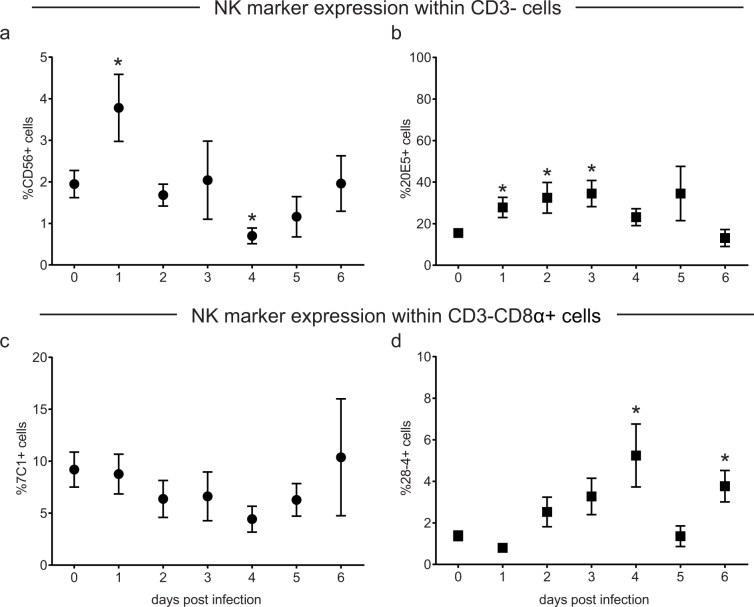
Different populations of NK cells were observed in the lung after LPAI virus infection. Frequencies of NK cells in the lung after LPAI virus infection were determined by flow cytometry using the NK-cell markers CD56 (a), 20E5 (b), 7C1 (c) and 28-4 (d). The frequency of marker positive cells within CD3- cells (A,B) and CD3-CD8α+ (c,d) are shown. Mean ± SEM of five infected birds per day and seventeen uninfected birds (birds from 0 dpi together with the uninfected birds from 1–6 dpi) are shown. Significant differences compared to the uninfected controls were analysed using Mann-Whitney tests and p < 0.05 is indicated by an asterisk.

The frequencies of NK cells in the lung after LPAI virus infection were determined by flow cytometry using markers that are known to be expressed on chicken NK cells; the chicken homologue of the human NK-cell marker CD56 and the previously described NK-cell markers 20E5, 7C1 and 28-4. Expression of the NK-cell markers was analysed within the CD3- population. The percentage of CD56+ NK cells increased after infection from 1.9 ± 0.3% in uninfected controls to 3.8 ± 0.8% at 1 dpi (p < 0.05, Fig. 4A). At 4 dpi, the percentage CD56+ NK cells temporarily dropped to levels lower than in uninfected controls (0.7 ± 0.2, p < 0.05). The frequency of 20E5+ NK cells increased continuously after infection until 3 dpi (34.5 ± 6.3%, p < 0.05 Fig. 4B). At 4 dpi, the percentage of 20E5+ NK cells decreased which resulted in levels similar to that of uninfected controls at 6 dpi.

In contrast to CD56 and 20E5, the percentage of 7C1+ NK cells (0.5 ± 0.1%) and 28-4+ NK cells (0.2 ± 0.04%) in the lung was rather low and did not change after infection (data not shown). Interestingly, these markers were readily expressed on the subpopulation of CD8α+ NK cells which are thought to represent a population of resting NK cells. The percentage of 7C1+ NK cells did not change during infection, while the percentage of 28-4+ NK cells increased during infection reaching levels upto 4% (4.6 ± 1.5% at 4 dpi; 4.3 ± 0.8% at 6 dpi; Fig. 4D). These differences in marker positive cells imply the presence of different populations of NK cells in the lung of chickens following LPAI virus infection.

### Infection with LPAI virus resulted in enhanced activation of lung NK cells

**Figure 5:**
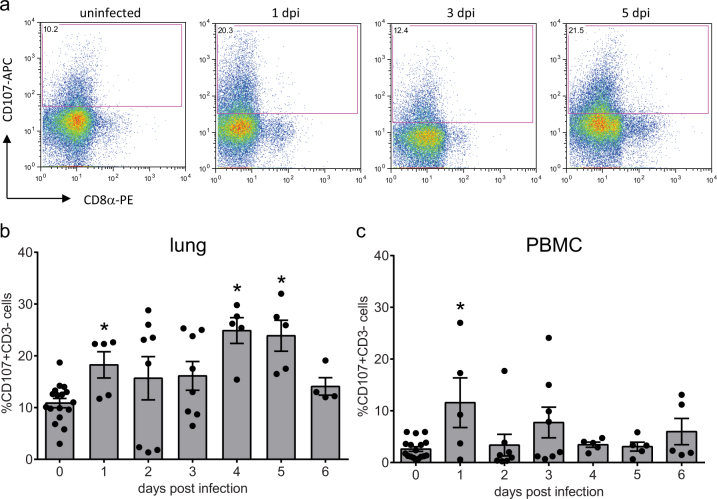
Infection with LPAI virus results in enhanced activation of lung NK cells. To study possible differences in NK-cell activation after infection with A/chicken/United Arab Emirates/99 H9N2 LPAI virus, cell surface expression of CD107 was analysed by flow cytometry. Representative FACS plots showing CD107 expression within lung CD3- cells from uninfected controls, and infected animals at 1, 3 and 5 dpi (a). Mean CD107 expression ± SEM is shown for lung (b) and PBMC (c) (n = 5 except for 2 dpi and 3 dpi n = 8). Significant differences compared to the uninfected controls (birds from 0 dpi together with the uninfected birds from 1–6 dpi) were analysed using Mann-Whitney tests and p < 0.05 is indicated by an asterisk.

To study the effect of LPAI virus infection on NK-cell activation, cell surface expression of CD107 was analysed. As virus detection in the spleen was limited, NK-cell activation induced by LPAI virus was only determined for lung and blood. Representative examples of CD107 expression on CD3- lung NK cells in uninfected controls and at 1, 3 and 5 dpi show that CD107 is only expressed at CD3- cell that do not express CD8α (Fig. 5A)Immediately after infection, the proportion of activated lung NK cells temporarily increased from 10.9 ± 0.9% in uninfected controls to 18.3 ± 2.5% at 1 dpi (p < 0.05). At 4 dpi the percentage of activated lung NK cells increased again to 24.9 ± 2.4%. The 4 dpi level was greater than the proportion of activated lung NK cells at 1 dpi (p < 0.05) and this high percentage of CD107 expressing cells continued until 5 dpi. At 6 dpi, the frequency of activated lung NK cells diminished to 14.1 ± 1.7% which was similar to the level observed in uninfected controls (Fig. 5B).

Although the percentage of activated NK cells in PBMC was much lower compared to that in lungs, a similar pattern was observed in the changes in expression early post-infection (Fig. 5C). The percentage of activated NK cells increased at 1 dpi from 2.6 ± 0.5% in uninfected samples to 11.6 ± 4.8% in infected birds (p < 0.05). The percentage of activated NK cells then decreased again to levels which were similar to uninfected controls.

### Gross pathology, and viral RNA levels upon H5N1 HPAI virus infection

Infection with LPAI virus resulted in increased activation of lung NK cells. The follow-up investigation was to determine the NK-cell response upon HPAI virus infection using the Eurasian H5N1 isolates tyTR05 and tyEng91. The analysis of NK-cell responses upon HPAI virus infection was limited by safety issues associated with working under Biosafety Containment Level 2 conditions. Therefore, *ex vivo* staining with NK-specific mAbs was not included in these HPAI experiments.

In contrast to the absence of lesions after LPAI infection, HPAI infection induced gross pathology lesions that were characteristic for AI virus infection (data not shown). Birds inoculated with tyTR05 H5N1 HPAI virus displayed moderate splenomegaly from 24 hpi, hyperplasia of the Bursa of Fabricius and hyperaemic lungs and thymus. Petechia in caecal tonsils was observed occasionally. Hyperaemic lungs and splenomegaly was also observed in chickens inoculated with tyENG91 H5N1 HPAI virus at 24 hpi.

**Figure 6:**
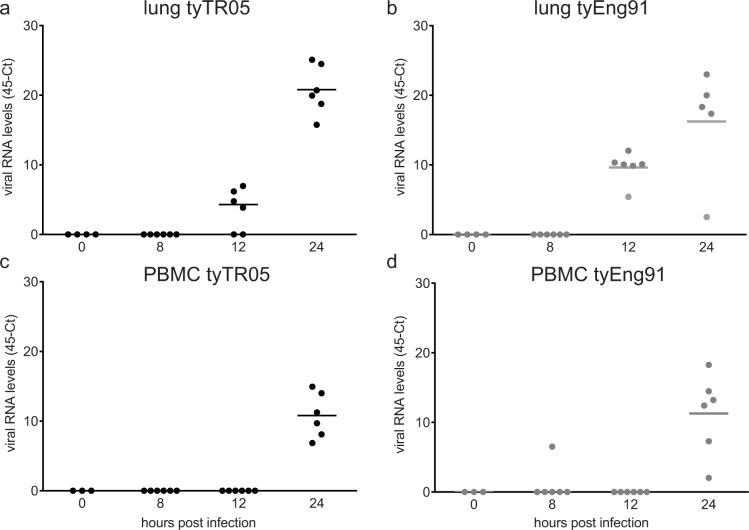
Viral load levels in lung and PBMC after infection with HPAI virus. Viral load was determined by qRT-PCR specific for the matrix gene of the influenza A virus. Relative expression values were normalized against 28S rRNA. Individual data are shown for lung tissue (a, b) and PBMC (c, d) following infection with A/turkey/Turkey/1/2005 (tyTR05) (a) or A/turkey/England/50-92/1991 (tyEng91) respectively (n = 6). The bar indicates the mean.

Twelve hours after infection with tyTR05, low viral load levels were observed in lungs of 4 out of 6 animals (45-Ct of 3.6 ± 1.2; Fig. 6A). At 24 hpi, virus was detected in lungs of all animals and increased to a 45-Ct level of 20.8 ± 1.4. Twelve hours after infection with tyEng91, virus was detected in the lungs of all animals and was higher compared to that observed after infection with tyTR05 (9.6 ± 0.9, p < 0.05, Fig. 6B) at the same time point. At 24 hpi the viral load was similar to levels observed 24 hpi with tyTR05 (16.2 ± 3.2). In PBMC, viral loads were only detected at 24 hpi (10.8 ± 1.3 tyTR05, Fig. 6C; 11.3 ± 1.3 tyEng91, Fig. 6D).

### H5N1 HPAI viruses do not induce activation of lung NK cells

**Figure 7:**
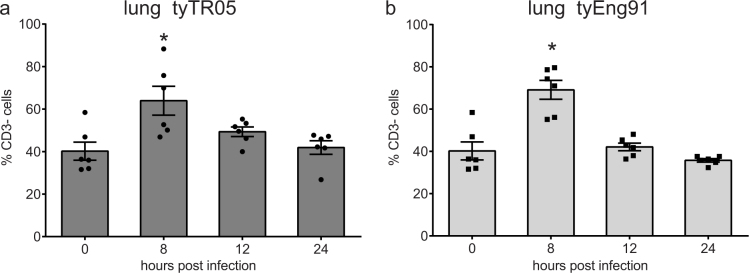
Lymphocytes numbers in the lungs after infection with HPAI virus. Percentages of lung CD3- cells within the live gate upon infection with tyTR05 (a) and tyEng91 (b) were analysed by flow cytometry. Mean ± SEM are shown (n = 6). Significant differences compared to the uninfected controls (0 hpi group) were analysed using Mann-Whitney tests and p < 0.05 is indicated by an asterisk.

Similar to what was observed after LPAI infection, infection with the Eurasian HPAI isolate tyTR05 resulted in a significant increase in the percentage of CD3- cells at 8 hpi compared to uninfected controls (64.0% ± 6.8 versus 40.2% ± 4.3, p < 0.05, Fig. 7C). Similar results were observed after infection with the Eurasian HPAI isolate tyEng91 (69.1% ± 4.5, p < 0.05, Fig. 7D). The increase in the percentage of CD3- cells was only transient, at 24 hpi with either tyTR05 or tyEng91 the percentage CD3- cells was similar to the percentage in uninfected controls.

**Figure 8:**
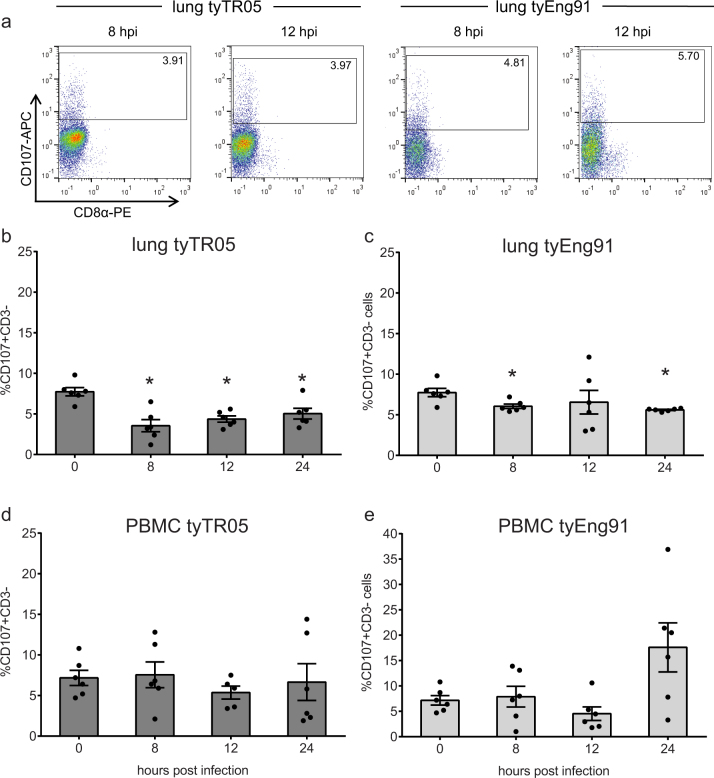
Decreased activation of lung NK cells after infection with HPAI virus. Representative FACS plots showing CD107 expression within lung CD3- cells from uninfected controls, and birds infected with tyTR05 and tyENG91 at 8 and12 hpi (a). Possible differences in NK-cell activation upon infection with the H5N1 HPAI viruses tyTR05 (b, d) and tyEng91 (c, e). Cell surface expression of CD107 in CD3- cells was analysed by flow cytometry using the same gating strategy as in Figure 7. Mean ± SEM are shown for lung (b, c) and PBMC (d, e) (n = 6). Significant differences compared to the uninfected controls (0 hpi group) were analysed using Mann-Whitney tests and p < 0.05 is indicated by an asterisk.

Representative examples of CD107 expression on CD3- lung NK cells in uninfected controls and chickens infected with tyTR05 and tyEng91 again show that CD107 is only expressed at CD3- cell that do not express CD8α (Fig. 8A).After infection with tyTR05, the percentage of activated NK cells in the lung decreased dramatically from 7.7 ± 0.5% in uninfected controls compared to 3.6 ± 0.7% at 8 hpi (p < 0.05). This decrease in the frequency of activated NK cells continued at 12 and 24 hpi (4.4 ± 0.4% and 5.0 ± 0.7% respectively; Fig. 8B). Infection with tyEng91 also resulted in a rapid decrease of activated NK cells (6.0 ± 0.3% at 8 hpi, p < 0.05) although the decrease was not as strong when compared to tyTR05 (Fig. 8C). At 24 hpi, the percentage of activated NK cells was still significantly lower compared to 0 hpi (5.6 ± 0.1% compared to 7.7 ± 0.5%, p < 0.05). In PBMC, the percentage of activated NK cells did not change upon infection with either tyTR05 (Fig. 8D) or tyEng91 (Fig. 8E). Thus, infection with Eurasian HPAI H5N1 virus results in decreased activation of lung NK cells.

## Discussion

Natural Killer cells and the activating NK-cell receptor NKp46 have been shown to play an important role in influenza-specific immunity^15^,^16^,^17^. Since avian influenza viruses use a distinct binding site on NKp46 compared to the human and swine influenza viruses and binding is dependent on α2,3-SA rather than α2,6-SA residues on the receptor^34^, the lack of NK-cell cytotoxicity upon binding of avian H5N1 virus to human NKp46 itself in the absence of NKG2D crosslinking may be due to differences in receptor specificity. We proposed an alternative mechanism and hypothesised that lack of NK-cell activation upon binding to NKp46 itself is an intrinsic property of these H5N1 HPAI viruses irrespective of the binding to α2,3- or α2,6-SA residues on the influenza specific NK-cell receptor NKp46.

To investigate this, NK-cell responses after infection were studied in chickens, which enabled us to use a system in which both viruses and NK cells are of avian origin. Studies on chicken NK cells have been hampered by the absence of specific mAbs for these cells. Originally chicken NK cells were defined as CD3-CD8α+ cells^35^. Similar results were observed for lung NK cells, which can be defined as T- and B-cell marker negative, and surface CD8α positive. It is most likely that this population of CD3-CD8α+ cells reflects resting NK cells, since NK-cell activation results in the down regulation of the CD8α chain^38^. This is again confirmed by our observation that CD107 is only expressed on cells that do not express the CD8α chain .When studying the frequencies of CD3-CD8α+ NK cells in the lung upon LPAI infection, higher levels of CD3-CD8α+ cells in the lung compared to uninfected controls were observed between 3 and 5 dpi. Also the percentage of CD3- cells was increased at 3 dpi. This increase may imply an influx of NK cells in the lung, similar to what has been described for mammalian species^40^. Since the absolute number of cells is not known, the increase in the percentage may also reflect a change in cell subsets in the lung, rather than an influx of NK cells. Immunohistochemistry is warranted to investigate the reason behind the increase in CD3- and CD3-CD8α+ cells in the lung.

Upon LPAI virus infection, diverse populations of NK cells were observed in the lung. The percentage of CD56+NK cells rapidly increased at 1 dpi, which may represent an influx of NK cells upon infection. The decrease in CD56 expression from 2 dpi onwards possibly reflects a down regulation upon activation similar to what has been described for human NK cells^41^. Clear differences were observed between lung cells expressing the markers 20E5, 7C1 and 28-4. Although all markers have been reported to be expressed on NK cells^38^,^39^ and the differences between the markers suggest the existence of distinct populations of NK cells, the antigens recognized by these markers are not yet known. This complicates a direct comparison between NK-cell populations in chickens and in humans or mice.

Infection with LPAI virus resulted in the enhanced activation of lung NK cells as determined by measuring cell surface expression of CD107. This enhanced activation was observed at 1 dpi and again at 4 and 5 dpi. We propose the following model; after infection, activation of lung NK cells occurs. NK cells can be activated directly via the interaction between AIV and a NK cell receptor, or indirectly via the production of cytokines like IL-12 and IL-18 by infected macrophages and dendritic cells. Despite this activation, viral load continues to increase. As reflected by the increase in the percentage of CD3-CD8α+ cells at 3 dpi, we hypothesise that resting NK cells enter the lung, subsequently become activated (as shown by the second peak in activation of lung NK cells at 4 and 5 dpi). Upon the second increase in NK-cell activation, a decrease in viral load is observed. At 6 dpi, viral load levels have reduced dramatically and the frequencies of NK cells in the lung and activation of lung NK cells have returned to levels observed in uninfected controls.

At 2 dpi and 3 dpi, analysis of activated lung NK cells showed two groups of birds: chickens with a high percentage of activated NK cells or chickens with a low percentage of activated NK cells. This can be partly explained by viral load levels; at both 2 and 3 dpi two birds show a lower viral load compared to the rest of the group.

Interestingly, the percentage activated NK cells in the uninfected controls differed between the LPAI and HPAI experiments. A possible explanation for this difference can be the fact that both experiments were performed in different institutes and therefore the chickens were housed in different animal facilities, Since a study in mice showed that the environment has a strong impact on the priming of NK cells^42^, housing under Biosafety Containment Level 2 conditions may result in less NK cell priming and less spontaneous degranulation which is reflected by the lower percentage of activated NK cells in the lung. This fits with our previous observation that uninfected SPF chickens that were housed in isolators also show a lower percentage of activated NK cells in the lung^43^ compared to the layer chickens that were used in this study.

In contrast to LPAI infection, infection with Eurasian H5N1 HPAI virus isolates did not result in activation of NK cells in the lung although similar amounts of virus were present. A decreased activation of NK cells compared to uninfected controls was found for two different H5N1 HPAI viruses and at multiple time points post infection. The decreased NK-cell activation may have several causes. Firstly, chicken NK cells express both activating and inhibitory NK-cell receptors^37^,^44^,^45^, so it is possible that chickens have influenza-specific NK-cell receptors similar to NKp46. Although both HPAI and LPAI viruses bind through α2,3-SA residues, differences in binding between HPAI and LPAI viruses may still occur, for example due to differences in glycosylation of HA^46^ or to binding to additional NK receptors. The difference in binding may lead to a different balance between activating and inhibiting receptors upon binding of LPAI or HPAI virus. Secondly, influenza virus has been reported to be able to infect NK cells, which results in NK-cell apoptosis and reduced NK-cell cytotoxicity^47^,^48^ and the number of NK cells is reduced in severely influenza infected patients^19^,^20^,^21^. It may be possible that HPAI viruses infect chicken NK cells, which could result in decreased NK-cell activation. Thirdly, infection with HPAI virus is characterized by a strong increase in viral load and a cytokine storm in humans and macaques^49^,^50^. Also in chickens a strong induction of cytokines upon HPAI infection has been reported, although the levels of cytokines induced seems to be dependent on the viral strain^51^,^52^. The overwhelming amount of virus and/or the presence of cytokines may produce an overstimulation of NK cells, resulting in NK-cell exhaustion. Lastly, in this study we used H9N2 and H5N1 viruses which are both endemic in poultry since their occurrence in the mid 90's^53^,^54^. To formerly proof that the lack of NK-cell activation upon H5N1 HPAI infection is an intrinsic property of HPAI viruses in general, experiments with an HPAI virus and its respective precursors should be performed.

In conclusion, we report enhanced activation of lung NK cells after infection with LPAI virus. Infection with HPAI virus results in decreased activation of lung NK cells indicating that decreased NK-cell activation may be one of the mechanisms contributing to the pathogenicity of H5N1 HPAI viruses. The mechanism behind this difference in activation of lung NK cells upon LPAI and H5N1 HPAI infections remains to be elucidated.

## Methods

### Animals

One-day old Lohmann Brown chickens were obtained from a commercial breeder. Chickens were housed in groups and fed *ad libitum* on commercial feed. At the age of 3 weeks, chickens were infected with either LPAI or HPAI virus. The LPAI H9N2 isolate A/chicken/United Arab Emirates/99 (kindly provided by Intervet/Merck Animal Health, Boxmeer, The Netherlands) was diluted in sterile PBS to a concentration of 1 × 10^6^ EID_50_/ml and chickens were inoculated intranasally and intratracheally (100 μl each). For each day from 0 and 6 dpi, 2 uninfected and 5 infected birds (8 infected birds at 2 dpi and 3 dpi) were killed by cervical dislocation and lungs, spleen and blood were collected. Pathology was performed to identify lesions associated with AIV or immune activation. In the analyses results from uninfected birds at 0 dpi are combined with the results from the uninfected birds at the other time points, since we did not observe any differences between these birds. Together these birds are referred to as “uninfected controls”. Tissue samples from lung (part L1)^55^, spleen and PBMC were collected in TRIzol reagent (Invitrogen, Bleiswijk, the Netherlands), snap-frozen in liquid nitrogen and stored in -80°C until RNA isolation was performed.

To obtain a single cell suspension, lung tissue was cut into small pieces and digested in RPMI containing 2 mg collagenase A from *Clostridium histolyticum* and 5 mg DNAse I isolated from bovine pancreas (Roche Applied Science, Almere, the Netherlands) for 30 min at 37°C, and homogenised using a 70 μM cell strainer (BD Biosciences, Franklin Lakes, NJ, USA). Spleens were also homogenised using a 70 μM cell strainer. Viable cells were isolated from lungs, spleen and blood by Ficoll-Paque density gradient centrifugation and the total number of lymphocytes in the lung was determined by counting the trypan blue negative cells. Cells were resuspended in IMDM medium supplemented with 8% heat inactivated FCS; 2% heat inactivated chicken serum, 100 U/ml penicillin, 100 μg/ml streptomycin and 2 mM glutamax (‘NK medium'; Gibco BRL, Paisly, United Kingdom) and were used directly in the NK-cell assays.

Alternatively, chickens were infected with H5N1 HPAI virus. Two different isolates were used which display different infection phenotypes in chickens: a new Eurasian lineage [A/turkey/Turkey/1/2005 (tyTR05)] and an old Eurasian lineage [A/turkey/England/50-92/1991 (tyEng91)] (B.Z. Löndt, personal communication). Virus was diluted in PBS to a concentration of 1 × 10^6^ EID_50_/0.1 ml and chickens were inoculated intranasally and intraocularly (50 μl each). Birds were killed at 0, 8, 12 and 24 hours post infection (hpi) (6 birds per time point) and lungs and blood were collected. In the HPAI experiment, uninfected birds are the birds killed at 0 hpi. Viable cells were isolated from these organs as described above. All animal experiments involving LPAI virus were approved by the Committee on Animal Experiments of the University of Utrecht (DEC 2008.II.01.010) and performed according the Dutch regulation on experimental animals. The experiments with the H5N1 HPAI viruses were performed at the Animal Health and Veterinary Laboratory Agencies according to the AHVLA committee for ethical studies and in accordance with the UK 1986 Animal Scientific Procedure Act and AHVLA code of practice for performance of scientific studies using animals (License number 70/7062).

### RNA isolation and quantitative real time PCR

Frozen tissue samples were thawed and lung and spleen samples were homogenised (Retsch Mixer Mill 301, Fisher Scientific, Landsmeer, The Netherlands) in TRIzol (Invitrogen). PBMC samples were homogenised using a syringe and a 21G needle. Total RNA was isolated according the TRIzol method using manufacturer's instructions followed by a DNAse treatment (RNase-Free DNase set, Qiagen Benelux, Venlo, the Netherlands). Purified RNA was eluted in 30 μl RNAse free water and the RNA was quantified by absorbance measurement at 280 nm. A maximum of 500 ng RNA was used for the cDNA generation using the iScript cDNA synthesis kit (Biorad laboratories, Veenendaal, the Netherlands). Real-time qRT-PCR was performed to detect the matrix gene of the influenza virus as previously described^56^. Briefly, a conserved fragment of the influenza matrix gene was amplified using the primers M-Fw (5′-CTTCTAACCGAGGTCGAAACGTA-3′), M-Rev (5′-CACTGGGCACGGTGAGC-3′), and the probe M (5′-FAM-CTCAAAGCCGAGATCGCGCAGA-3′-TAMRA) in combination with the Taqman Universal PCR mastermix (Applied Biosystems, Nieuwerkerk aan de IJssel, The Netherlands) on a MyiQ Single color Realtime PCR Detection system (Biorad). Relative expression values were normalized against 28S rRNA, as previously described by Kaiser et al^57^. Mean threshold cyle values (Ct) were determined based on triplicates and results are shown as 45-Ct values, which are calculated by subtracting the experimental Ct value from the max Ct value.

### Flow cytometry

Characterization of avian NK cells in lung was performed by flow cytometry using co-stainings with mouse-anti-chicken CD3 (CT3; IgG1,), mouse anti-chicken-CD8α- PE mAbs (CT8, IgG1), mouse-anti-chicken-Bu-1 (AV20, IgG1), mouse-anti-chicken TCR γδ (TCR1, IgG1), mouse-anti-chicken TCR αβ-Vβ1 (TCR2, IgG1) and mouse-anti-chicken TCR αβ-Vβ2 (TCR3, IgG1) on lung cells of an uninfected bird. All antibodies were obtained from Southern Biotec, Birmingham, AL, USA. Cells were stained with the antibodies for 20 min at 4°C.

Proportions of NK cells were analysed by flow cytometry using markers that are known to be expressed on chicken NK cells; the chicken homologue of the human NK-cell marker CD56^58^ and the previously described NK-cell markers 20E5, 7C1^38^ and 28-4^39^. Since some of the NK-cell markers are also expressed on T cells^38^, staining with NK-cell markers was combined with anti-CD3 mAb. Cells were stained with hybridoma supernatants^38^ for 30 min at 4°C, followed by a goat-anti-mouse IgG secondary Ab (Southern Biotec) for 20 min 4°C. Normal mouse serum was used to block non-specific binding followed by staining with anti- CD3-FITC and anti-CD8α-PE mAbs.

CD107 expression was analysed by staining with a mouse-anti-ChCD107 biotinylated mAb^38^, followed by a fluorochrome-labelled streptavidin secondary antibody (BD Biosciences). Alternatively, an APC-conjugated anti-CD107 antibody was used. In all experiments, staining with anti-CD107 was combined with anti-CD3 and anti-CD8α mAbs to exclude T cells from the analyses.

Prior to flow cytometry, 7-Amino-Actinomycin D (7AAD; BD Biosciences) or the live/dead® fixable violet cell stain (Invitrogen) was used for exclusion of dead cells. At least 50,000 cells in the live gate were acquired using a FACS Calibur flowcytometer (BD Biosciences). Samples from the experiments with the H5N1 HPAI viruses were acquired using a MACSQuant® Analyser (Miltenyi Biotec, Bergisch Gladbach, Germany). Data were analysed using the software program FlowJO (Threestar Inc, Ashland, OR, USA) or FACS DIVA software (BD Biosciences).

### CD107 assay

The CD107 assay to study NK-cell activation was essentially carried out as described previously^38^. Briefly, cells isolated from lung, spleen and blood were resuspended in NK medium at a concentration of 1 × 10^6^ cells/ml. Cells were cultured in the presence of 1 μl/ml Golgistop (BD Biosciences) and anti-ChCD107 mAb during 4 hours at 37°C, 5% CO_2_. After incubation, cells were washed in PBS supplemented with 0.5% BSA, and stained with anti-chicken-CD3-FITC and anti-chicken-CD8α-PE mAbs and analysed by flow cytometry. After staining cells from animals infected with HPAI virus, cells were fixed using a 4% paraformaldehyde (Merck, Darmstadt, Germany) for 10 minutes at room temperature. The cells were then washed once in PBS supplemented with 0.5% BSA and flow cytometry was performed as described above.

### Statistical analyses

Non-parametric statistical tests were used when the assumption of normally distributed data were not met. Differences between the groups were analysed using Mann-Whitney U tests. A p-value of < 0.05 was considered statistically significant. All statistical analyses were performed using the software program SPSS 16.0 (SPSS Inc, Illinois, USA).

## Author Contributions

C.J. designed the study, generated data of both LPAI and HPAI experiments, analysed the data and wrote the manuscript. E.d.G. was involved in the design of the study, generated data and analysed data of the LPAI experiment. D.v.H. and P.v.d.H. were involved in generating data of the LPAI experiments as well as data analysis. B.L. was involved in the design of the study, supplied the HPAI viruses, generated the HPAI data and wrote the manuscript. S.G. supplied reagents, analysed the HPAI data and wrote the manuscript. T.G. supplied materials to analyse NK cell frequencies and function and was involved in writing the manuscript. W.v.E. was involved in data analysis and writing the manuscript. S.B. was involved in the design of the study, supplied HPAI viruses and wrote the manuscript. L.V. generated data of both LPAI and HPAI experiments, was involved in data analysis and wrote the manuscript.
